# Association of serum ADMA, SDMA and L-NMMA concentrations with disease progression in COVID-19 patients

**DOI:** 10.11613/BM.2023.010701

**Published:** 2022-12-15

**Authors:** Adnan Haşimi, Özlem Doğan, Ceyhan Ceran Serdar, Muhittin A. Serdar

**Affiliations:** 1 Department of Medical Biochemistry, Faculty of Medicine, Medipol University, Istanbul, Turkey; 2 Department of Biochemistry, Ankara University School of Medicine, Ankara, Turkey; 3 Medical Biology and Genetics, Faculty of Medicine, Ankara Medipol University, Ankara, Turkey; 4 Department of Medical Biochemistry, Acibadem Mehmet Ali Aydinlar University, Istanbul, Turkey

**Keywords:** SARS-CoV-2, asymmetric dimethylarginine (ADMA), N-monomethyl-1 -arginine (L-NMMA), prognosis

## Abstract

**Introduction:**

This study determines and compares the concentrations of arginine and methylated arginine products ((asymmetric dimethylarginine (ADMA), symmetric dimethylarginine (SDMA), n-monomethyl-1-arginine (L-NMMA) and homoarginine (HA)) for assessment of their association with disease severity in serum samples of COVID-19 patients.

**Materials and methods:**

Serum arginine and methylated arginine products of 57 mild-moderate and 29 severe (N = 86) COVID-19 patients and 21 controls were determined by tandem mass spectrometry. Moreover, the concentrations of some of the routine clinical laboratory parameters -neutrophil lymphocyte ratio (NLR), C-reactive protein, ferritin, D-dimer, and fibrinogen measured during COVID-19 follow-up were also taken into consideration and compared with the concentrations of arginine and methylated arginine products.

**Results:**

Serum ADMA, SDMA and L-NMMA were found to be significantly higher in severe COVID-19 patients, than in both mild-moderate patients and the control group (P < 0.001 for each). In addition, multiple logistic regression analysis indicated L-NMMA (cut-off =120 nmol/L OR = 34, 95% confidence interval (CI) = 3.5-302.0, P= 0.002), CRP (cut-off = 32 mg/L, OR = 37, 95% CI = 4.8-287.0, P < 0.001), and NLR (cut-off = 7, OR = 22, 95% CI = 1.4-335.0, P = 0.020) as independent risk factors for identification of severe patients.

**Conclusions:**

The concentration of methylated arginine metabolites are significantly altered in COVID-19 disease. The results of this study indicate a significant correlation between the severity of COVID-19 disease and concentrations of CRP, NLR and L-NMMA.

## Introduction

The Coronavirus Disease 2019 (COVID-19), caused by severe acute respiratory syndrome coronavirus 2 (SARS-CoV-2), is commonly either seen as an asymptomatic disease or manifests itself as a mild respiratory tract infection. Nevertheless approximately 1/5 of the patients still require hospitalization, some in need of respiratory support or intensive care, others even resulting in exitus (*1*, *2*).

As multifactorial, multiplexed, and multicentric data regarding this disease rapidly accumulates, significant efforts have been made to unveil prognostic biomarkers. For this purpose, numerous studies conducted risk analyses of different parameters, demonstrating their association with disease severity and mortality rate. In this context, numerous clinical laboratory parameters *(i.e.*, neutrophil/lymphocyte ratio (NLR), platelet count, C-reactive protein (CRP), ferritin, lactate dehydrogenase (LDH), procalcitonin (PCT), troponin, interleukin-6 (IL-6), D-dimer, liver enzymes) have been scrutinized by various research groups. In addition, the number of studies examining other biochemical and molecular parameters is rapidly increasing to elucidate the physiopathology of the disease (*3*-*7*). While numerous studies have been carried out to repurpose parameters such as mid-regional pro-adrenomedullin (MR-pro-ADM), monocyte distribution width, or various micro RNAs (miRNAs) as novel biomarkers for predicting the risk of COVID-19 severity, limitations experienced with each of them indicate that there is still need for the identification of a better biomarker (*8*, *9*).

Arginine, as one of the key amino acids required in protein and creatine-synthesis, serves several other important functions in the cell. While it is converted into nitric oxide (NO) and citrulline through the action of nitric oxide synthase (NOS), it is metabolized into ornithine and urea through the action of arginase-I (ARG-I) or arginase-II (ARG-II), further facilitating the synthesis of polyamines, proline, and glutamate. In addition, arginine serves as a precursor for molecules such as asymmetric dimethylarginine (ADMA), symmetric dimethylarginine (SDMA), N-monomethyl-1-arginine (L-NMMA) (*10*-*12*).

Methylation of the arginine residue on a protein is catalysed by protein N-arginine methyltransferase (PRMT) enzymes, whereby a methyl group from S-adenosyl-l-methionine (SAM) is transferred to the terminal nitrogen on the guanidinium moiety of arginine. Depending on the number (either one or two) and the position of the transferred methyl groups, the arginine residues are converted into monomethylarginine residues (Rme1), asymmetric dimethylarginine residues (Rme2a) or symmetric dimethylarginine residues (Rme2s). Proteolytic degradation of arginine methylated proteins produces L-NMMA, ADMA, and SDMA metabolites depending on the type of methylarginine residues (Rme1, Rme2a, and Rme2s, respectively) (*13*).

While ADMA and L-NMMA reduce NO production by directly inhibiting NOS enzymes, SDMA acts by blocking the entrance of its substrate, arginine, into the cells. Consequently, an increase in the concentrations of ADMA and related metabolites (SDMA, L-NMMA) and the arginine/ADMA ratio were shown to be highly significant in elucidating inflammatory responses, renal and neurodegenerative disorders, and especially cardiovascular diseases. The relationship between the prognosis or mortality of various diseases and serum concentrations of ADMA and SDMA was demonstrated in many disorders (*14*). L-arginine metabolites (ADMA, SDMA, *etc.*) were also documented to be associated with hypoxia and septic shock (*15*, *16*).

In conjunction with the importance of proper endothelial cell functions in the course of COVID-19 disease, the significance of dysregulation in NO metabolism is frequently discussed. Angiotensin-converting enzyme-2 (ACE2), as the receptor of SARS-CoV-2, is an important modulator of NO release. Metabolites of L-arginine, ADMA, and SDMA were documented to cause endothelial NO inhibition (*17*, *18*). They induce NO synthase with inflammatory cytokines released during COVID-19 infection. On the other hand, ADMA and SDMA affect the immune response by inhibiting NO synthase activity. It has been suggested that high concentrations of ADMA and SDMA are associated with an increase in the severity of the disease and the prevalence of mortality during COVID-19 (*19*).

This study aims to address the relationship between disease severity of COVID-19 cases and arginine concentration, concentrations of methylated arginine metabolites (ADMA, SDMA, L-NMMA, homoarginine), and the concentrations of routine clinical laboratory parameters (such as NLR, CRP, ferritin, D-dimer, fibrinogen).

## Materials and methods

Eighty-six patients positive for COVID-19 and age-matched 21 control cases were included in this case-control study. Pharyngeal and nasal swab samples of all COVID-19 patients were analysed by real-time reverse transcriptase-polymerase chain reaction (RT-PCR) method in accordance with WHO interim guidance (*20*). Ethical approval for all the procedures was obtained from Clinical Research Ethics Committee (İstanbul Medipol University, Clinical Research Ethics Committee, March 11, 2021, E-10840098-772.02-854), and informed consent was collected from all participants. The study was conducted between June and December 2021 at Istanbul Medipol University.

According to the classification of the most recent COVID-19 (SARS-CoV-2 Infection), the Adult Patient Treatment Guideline prepared by the Turkish Ministry of Health COVID-19 patients are divided into two groups: 1) mild-to-moderate and 2) severe (*21*). Patients exhibiting mild/moderate pneumonia symptoms such as fever, sore throat, cough, peripheral capillary oxygen saturation (SpO2) > 90 mmHg at room temperature, breathing rate < 30/min, and muscle/joint pain were grouped as ‘mild-to-moderate’. Patients which are classified as ‘severe’ are those (a) who exhibit severe pneumonia symptoms such as fever, sore throat, cough, muscle/joint pain, with breathing rate > 30/min and require hospitalization, or (b) who are clinically compatible yet demonstrate SpO2 < 90 mmHg with breathing rate < 30/min and need hospitalization or (c) whose computerised tomography (CT) scan results indicate bilateral diffuse pneumonia findings. Since impaired urination has a disruptive influence on concentrations of arginine and methylated arginine metabolites, patients with kidney disease, glomerular filtration rate (GFR) < 60 mL/min, and proteinuria were excluded from the study.

Baseline comorbidity (hypertension, diabetes mellitus, chronic obstructive pulmonary disease, obesity, thyroid disorders, rheumatoid arthritis, systemic lupus erythematosus, heart disease, *etc.*) information was obtained from the patient files in which anamnesis and physical examination information were recorded. Patients with renal disorders were excluded from this study as it may alter the excretion of arginine metabolites.

The control group consisted of individuals of similar age who were admitted to the hospital for different reasons and whose PCR test was found to be negative.

Methods and instruments used to analyse routine clinical laboratory parameters (NLR, creatinine, CRP, LDH, IL-6, ferritin, D-dimer, fibrinogen) are listed in Table 1.

**Table 1:** Methods and instruments used to analyse routine clinical laboratory parameters

**Analyte**	**Analyser**	**Country**	**Method**
Neutrophil lymphocyte, ratio (NLR)	Sysmex XN 9000	Sysmex Corp, Kobe, Japan	Fluorescence flow cytometry
D-dimer (ng/mL)	ACL TOP 700	Instrumentation Laboratory, Paris, France	Immunoturbidimetric
Fibrinogen (g/L)	ACL TOP 700	Instrumentation Laboratory, Paris, France	Chromogenic
Total protein (g/L)	Roche Cobas 8000	Roche Diagnostic, Mannheim, Germany	Biuret
Albumin (g/L)	Roche Cobas 8000	Roche Diagnostic, Mannheim, Germany	Colorimetric BCG
C reactive protein (mg/L)	Roche Cobas 8000	Roche Diagnostic, Mannheim, Germany	Immunoturbidimetric
Creatinine (µmol/L)	Roche Cobas 8000	Roche Diagnostic, Mannheim, Germany	Modified Jaffé
Ferritin (µg/L)	Roche Cobas e411	Roche Diagnostic, Mannheim, Germany	ECLIA
BCG – Bromocresol green. ECLIA – Electrochemiluminescence immunoassay.			

Methylated arginine metabolites were quantified utilizing a slightly modified version of the method published by Di Gangi *et al.* (*22*). In this modified method, the samples were applied onto a Phenomenex (Torrance, CA, USA) 75 x 4.6 mm x 4 µm polar-RP column and run on Dionex HPLC, and Access MAX liquid chromatography with tandem mass spectrometry (LC-MS/MS) (Thermo Scientific, USA) devices, using “40 mM ammonium formate - 3% formic acid” as mobile phase A and “acetonitrile” as mobile phase B. The flow rate was 300 uL/min, and the sequence of the mobile phases applied onto the column was as follows: isocratic 10% B for the first two min, linear-gradient 10-30% B during t: 2-8 min, isocratic 30% B during t: 8-11 min, isocratic 10% B during t: 11-20 min.

Multiple reaction monitoring (MRM) parameters (m/z ratios of precursor and daughter ions) and optimal collision energy (CE) values for each analyte were determined in preliminary fragmentation studies through continuous infusion of 40 mmol/L stock solutions of each analyte onto the mass spectrometer.

Ratios of m/z of precursor and daughter ions and CE (in parenthesis) for each analyte were determined as follows: 259.3-214.0 (25 V), 266.6-221.0 (15 V), 259.3-228.0 (15 V), 245.3-70.2 (15 V), 231.3-70.1 (15 V), 245.2-84.2 (24 V), for ADMA, ADMA-D7 (internal standard), SDMA, L-NMMA, arginine, homoarginine, respectively. Additional conditions were as follows: capillary temperature: 210°C; vaporizer temperature: 350°C; sheath gas: 30 Arb, Aux gas: 10 Arb, spray voltage: 3000 V; and polarity: (+).

Serial dilutions of the respective stable isotypes were used as calibration standards. Concentration of the standards were 0.05, 0.1, 0.5, 1, 2.4 µmol/L for ADMA, SDMA and homoarginine; 5, 10, 50, 100, 200, 400 nmol/L for L-NMMA; and 5, 10, 50, 100, 200, 400 µmol/L for arginine.

Intra-, inter-assay coefficient of variation (%) and recovery (%) values were found to be < 6.4, < 10.2% and 103% for ADMA; < 7.1, < 12.1% and 96% for SDMA; < 7.8, < 14.2% and 96% for L-NMMA; < 5.1, < 7.8% and 98% for arginine; and < 6.7, < 11.6% and 105% for homoarginine, respectively.

Limit of quantitation (LoQ) values were determined to be 0.02 µmol/L, 0.04 µmol/L, 9.3 nmol/L, 3.1 µmol/L, and 0.05 µmol/L for ADMA, SDMA, L-NMMA, arginine, and homoarginine, respectively.

Aliquots of 200 µL, from each serum sample, were placed in a fresh tube and mixed with 100 µL ADMA-D7 (1 µM) internal standard and 1 mL methanol. The tubes were vortexed for 1 minute and centrifuged at 13,000 rpm for 10 minutes at room temperature. The supernatants were then transferred to fresh tubes, and the solvent was evaporated under nitrogen at 65°C. Subsequently, 200 µL of the derivatization solution prepared by mixing 19 volumes of butanol with 1 volume of acetyl chloride was added. The samples were incubated at 65°C for 30 minutes for derivatization. After incubation, the samples were evaporated under nitrogen. Pellet remnants were dissolved with 200 µL of “10% methanol - 0.1% formic acid” solution. Volume of 20 µL of the dissolved samples were transferred to autosampler vials for LC–MS/MS analysis. Samples that were separated chromatographically in the analytical column were then transferred to the mass spectrometer. Selected ions (precursor ions) were broken down into fragments (daughter ions) in the collision cell of the tandem MS, enabling more definitive measurements.

### Statistical analysis

The conformity of the data to distribution for continuous variables was evaluated using the Shapiro-Wilks test. One-way ANOVA and Kruskal Wallis were applied for comparisons of laboratory parameters between patients and control groups. Bonferroni and Tukey tests were performed for *post-hoc* analysis (Table 2). Association between two quantitative variables was determined by Pearson correlation if the data were normally distributed and by Spearman correlation if the data did not follow a normal distribution. The correlation coefficients (r) were interpreted as “weak or no linear relationship” for r-values between 0 to 0.25 (or - 0.25 to 0); “fair degree of linear relationship” for r-values between 0.25 to 0.5 (or - 0.5 to - 0.25); “moderately strong linear relationship” for r-values between 0.50 to 0.75 (or - 0.75 to - 0.5); “very strong linear relationship” for r-values > 0.75 (or < - 0.75); or “perfect linear relationship” for r-value = 1 (or = - 1) (*23*).

**Table 2:** Demographic and laboratory findings of mild-moderate and severe COVID-19 patients and control cases

	**Control** **(N = 21)**	**Mild-Moderate** **(N = 57)**	**Severe** **(N = 29)**	**P**
Age (years)	57 (25-72)	40 (18-79)	60 (18-89)*	< 0.001^‡^
Male (proportion)	7 (0.33)	35 (0.61)	12 (0.41)	0.046^║^
Comorbidity (total)	14	38	22	0.657^║^
Number of patients with one / two / more than two comorbidities	8 / 5 / 1	19 / 14 / 5	5 / 12 / 6	0.146^║^
Arterial hypertension	9	31	18	/
Diabetes mellitus	3	9	5	/
COPD	2	7	3	/
Obesity	3	7	4	/
Thyroid disorders	1	3	2	/
Autoimmune disorders (RA, SLE)	1	2	1	/
Heart diseases	2	7	4	/
Others	2	2	2	/
Creatinine (µmol/L)	79 ± 19	86 ± 16	88 ± 22	0.214^‡^
NLR	1.5 (1.3-1.8)	2.1 (1.61-3.91)*	9.5 (3.4-15.5)^*†^	< 0.001^§^
D-dimer (mg/L)	55 (27-74)	163 (111-254)*	1190 (439-3227)^*†^	< 0.001^§^
CRP (mg/L)	2.0 (0.9-2.6)	7.5 (3.1-20.6)*	122 (54.6-197)^*†^	< 0.001^§^
Fibrinogen (g/L)	1.27 (0.87-1.70)	3.35 (2.86-4.38)*	5.68 (2.87-6.98)^*†^	< 0.001^§^
Ferritin (µg/L)	44 (22-80)	59 (23 - 171)	216 (92 -515)^*†^	0.001^§^
Albumin (g/L)	44 ± 2	41 ± 4*	34 ± 8^*†^	< 0.001^‡^
Total protein (g/L)	75 ± 3	70 ± 5*	63 ± 9^*†^	< 0.001^‡^
Arginine (µmol/L)	74 ± 20	76 ± 21	78 ± 31	0.940^‡^
L-NMMA (nmol/L)	83 ± 27	101 ± 46	156 ± 58^*†^	< 0.001^‡^
Homoarginine (µmol/L)	1.54 ± 0.57	1.41 ± 0.62	1.30 ± 0.73	0.296^‡^
SDMA (µmol/L)	0.55 ± 0.08	0.56 ± 0.21	0.93 ± 0.46^*†^	< 0.001^‡^
ADMA (µmol/L)	1.18 ± 0.19	1.21 ± 0.35	1.66 ± 0.39^*†^	< 0.001^‡^
Duration of hospitalization (days)	/	5 (2-9)	16 (10-25)	0.001
*Comparison of test results with the control group (P < 0.05). ^†^Comparison of test results with mild-moderate group (P < 0.05). ^‡^ANOVA tests and *post-hoc* Tukey tests are applied for parameters with normal distribution; results are presented as mean ± SD for parameters with normal distribution. ^§^Kruskal Wallis Tests and *post-hoc* Bonferroni correction is applied for the parameters which do not follow normal distribution; results are presented as median (25-75%). ^║^Chi-squared test is applied for qualitative parameters. COPD – Chronic obstructive pulmonary disease. RA – Rheumatoid arthritis. SLE – Systemic lupus erythematosus. NLR – Neutrophil lymphocyte ratio. CRP – C-reactive protein. L-NMMA – N-monomethyl-L-arginine. SDMA – Symmetrical dimethyl arginine. ADMA – Asymmetric dimethyl arginine.				

Receiver operating characteristic (ROC) analysis was performed for each parameter that was found to be different in the group comparison analysis and the most appropriate cut-off values for these parameters were determined. A univariate logistic regression model was used to identify the independent predictors associated with COVID-19 severity. Subsequently, multivariate regression analysis was also performed to determine the Odd Ratios (OR). The logistic transformation was used for the regression analyses of parameters that did not show normal distribution (NLR, CRP, D-dimer, fibrinogen, ferritin). Analyses were conducted using SPSS Statistics 24 (IBM, NY, USA). Value P < 0.05 was considered statistically significant.

## Results

Demographic data, comorbidity and laboratory findings of mild-moderate and severe COVID-19 patients and the control group, together with the statistical analysis results, are presented in Table 2.

**Figure 1:**
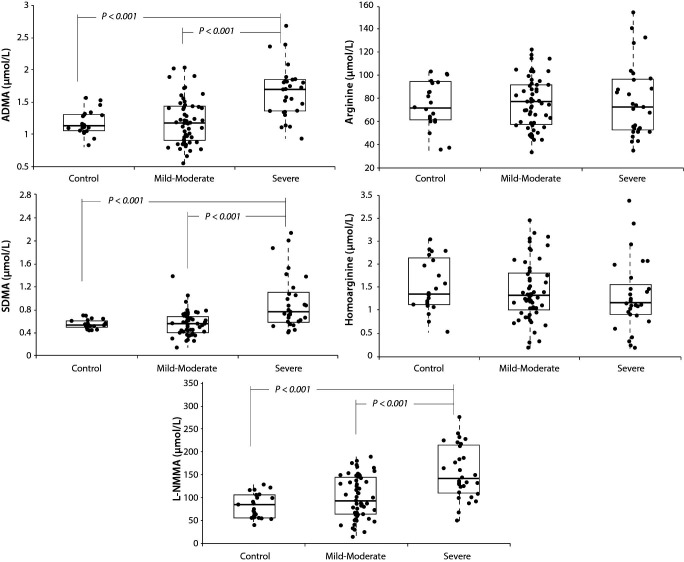
Comparison of arginine, homoarginine, L-NMMA, ADMA, and SDMA concentrations COVID-19 patients and the control group. L-NMMA – N-monomethyl-l-arginine. ADMA – Asymmetric dimethyl arginine. SDMA – Symmetrical dimethyl arginine.

In the severe patients, ADMA, SDMA, and L-NMMA were found to be significantly higher than in both the mild-moderate patients and the control group (Table 2 and Figure 1).

Concentrations of NLR, CRP, D-dimer, fibrinogen, ferritin, albumin, and total protein of the control group were significantly different than their counterparts in both mild-moderate and severe COVID-19 patients. Concentrations of these parameters were also significantly different between mild-moderate and severe COVID-19 patients (Table 2). The duration of hospitalization was found to be higher in the severe group, as expected.

In the study group, no statistical difference was found between severe COVID-19 patients and other groups in terms of number of patients with one / two / more than two comorbidities.

Results of the correlation analyses among all parameters are presented in Table 3 and indicate that methyl arginine metabolites are positively correlated with each other in the COVID-19 context. Concentrations of SDMA and ADMA were found to have fair degree of linear correlation with D-dimer, CRP, ferritin, total protein, and albumin. On the other hand, while D-dimer and total protein concentrations had a fair degree of linear correlation with L-NMMA concentration, they had a weak correlation with CRP and NLR concentrations.

**Table 3:** Results of the correlation analysis between methylated arginine metabolites and other parameters

**Analyte**		**Age**	**Arginine**	**L-NMMA**	**Homoarginine**	**SDMA**	**ADMA**
Arginine	r	0.06	/	/	/	/	/
	P	0.542	/	/	/	/	/
L-NMMA	r	0.15	0.26	/	/	/	/
	P	0.113	0.020	/	/	/	/
Homoarginine	r	- 0.01	0.48	0.11	/	/	/
	P	0.937	< 0.001	0.310	/	/	/
SDMA	r	0.20	0.27	0.56	0.02	/	/
	P	0.039	0.005	< 0.001	/	/	/
ADMA	r	0.24	0.27	0.68	0.01	0.72	/
	P	0.012	0.005	< 0.001	0.949	< 0.001	/
NLR	r	0.31	-0.09	0.23	- 0.21	0.28	0.31
	P	0.001	0.346	0.017	0.030	0.003	0.001
D-dimer	r	0.32	0.11	0.37	-0.10	0.35	0.37
	P	0.001	0.262	< 0.001	0.305	< 0.001	< 0.001
CRP	r	0.32	0.01	0.21	- 0.12	0.29	0.26
	P	0.001	0.946	0.032	0.207	0.002	0.007
Fibrinogen	r	0.13	0.10	0.18	- 0.12	0.10	0.11
	P	0.174	0.317	0.064	0.217	0.313	0.263
Ferritin	r	0.36	0.04	0.14	- 0.03	0.31	0.30
	P	< 0.001	0.731	0.185	0.785	0.004	0.038
Duration of hospitalization	r	0.36	0.06	0.24	- 0.22	0.10	0.26
	P	0.001	0.559	0.406	0.134	0.357	0.018
Total protein	r	- 0.14	0.07	0.26	0.20	- 0.30	- 0.26
	P	0.162	0.481	0.007	0.048	0.002	0.008
Albumin	r	- 0.31	0.001	- 0.18	0.27	- 0.29	- 0.25
	P	0.001	0.990	0.077	0.007	0.003	0.010
L-NMMA – N-monomethyl-L-arginine. SDMA – Symmetrical dimethyl arginine. ADMA – Asymmetric dimethyl arginine. NLR – Neutrophil lymphocyte ratio. CRP – C-reactive protein.							

**Figure 2:**
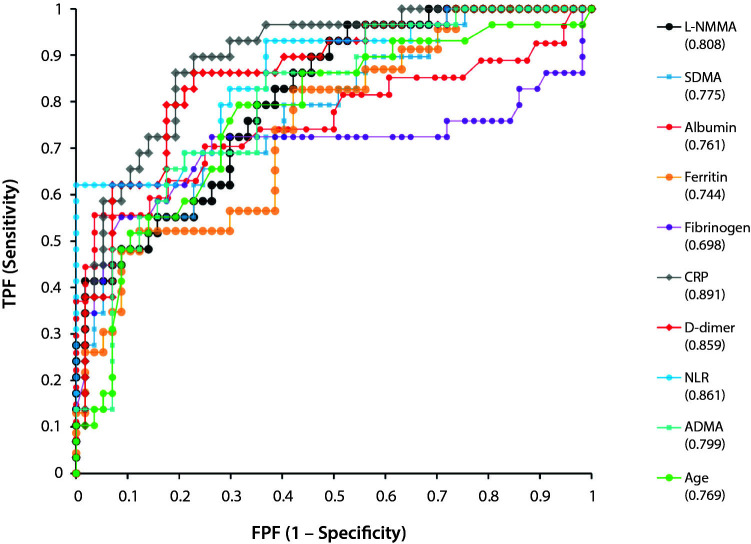
A receiver operating characteristic curve (ROC) analysis of all parameters according to severity of COVID-19. TPF – True positive fraction. FPF – False positive fraction. L-NMMA – N-monomethyl-l-arginine. SDMA – Symmetrical dimethyl arginine. CRP – C-reactive protein. NLR – Neutrophil lymphocyte ratio. ADMA – Asymmetric dimethyl arginine.

Receiver operating characteristic analyses were performed to determine the area under the curve (AUC), optimal cut-off value, sensitivity and specificity values of age, ADMA, SDMA, L-NMMA, NLR, CRP, D-dimer, fibrinogen, ferritin, total protein, albumin in correlation with COVID-19 severity (Table 4 and Figure 2). All parameters were categorized accordingly, and their univariate logistic regression analyses were performed (Table 5). While univariate logistic regression analyses indicated age, gender and biochemical parameters listed on Table 4 as statistically significant parameters associated with COVID-19 severity; multivariate regression analysis only indicated L-NMMA (cut-off = 120 nmol/L, OR = 34, 95% confidence interval (CI) = 3.5-255.0, P = 0.002), CRP (cut-off = 32 mg/L, OR = 37, 95% CI = 4.8-302.0, P < 0.001), and NLR (cut-off = 7, OR = 22, 95% CI = 1.4-335.0, P = 0.020) as independent risk factors.

**Table 4:** The optimum cut-off, area under curve (AUC), sensitivity and specificity values of all parameters in severe COVID-19 disease (comparison of mild to moderate and severe patients)

	**Cut-off**	**AUC**	**95% CI for AUC**	**Sensitivity (%)**	**Specificity (%)**
Age (years)	> 46	0.77	0.66 to 0.88	79	68
ADMA (µmol/L)	> 1.29	0.80	0.70 to 0.90	86	63
L-NMMA (nmol/L)	> 120	0.81	0.72 to 0.90	79	65
SDMA (µmol/L)	> 0.75	0.77	0.67 to 0.88	52	89
Ferritin (µg/L)	> 74	0.74	0.63 to 0.86	83	58
Fibrinogen (g/L)	> 5.4	0.70	0.55 to 0.85	55	91
CRP (mg/L)	> 32	0.89	0.82 to 0.96	86	81
D-dimer (mg/L)	> 257	0.86	0.78 to 0.94	86	77
NLR (ratio)	> 7.0	0.86	0.78 to 0.95	62	95
Total protein (g/L)	≤ 61	0.74	0.60 to 0.87	48	96
Albumin (g/L)	≤ 34	0.76	0.63 to 0.89	56	96
ADMA – Asymmetric dimethyl arginine. L-NMMA – N-monomethyl-L-arginine. SDMA – Symmetrical dimethyl arginine. CRP – C-reactive protein. NLR – Neutrophil lymphocyte ratio.					

**Table 5:** Univariate and multivariate binary logistic regression analyses among parameters related to COVID-19 disease severity (comparison of mild to moderate and severe patients)

	**Univariate analysis**			**Multivariate analysis**		
	P	OR	95% CI for OR	P	OR	95% CI for OR
Age	< 0.001	8.3	2.8 to 23.9			
Gender (male)	0.018	3.1	1.2 to 7.7			
ADMA	< 0.001	10.7	3.2 to 35.1			
SDMA	< 0.001	7.6	2.6 to 22.4			
L-NMMA	< 0.001	7.1	2.4 to 20.2	0.002	34	3.5 to 302.0
CRP	< 0.001	26.1	7.5 to 90.6	< 0.001	37	4.8 to 287.0
NLR	< 0.001	25.5	6.4 to 101.0	0.020	22	1.4 to 335.0
D-dimer	< 0.001	21.1	6.2 to 71.9			
Ferritin	0.002	6.5	1.9 to 21.6			
Fibrinogen	< 0.001	12.8	3.9 to 41.3			
Total protein	< 0.001	21.6	4.3 to 107.0			
Albumin	< 0.001	31.7	6.4 to 156.0			
Cl – confidence interval. OR – Odds ratio. ADMA – Asymmetric dimethyl arginine. SDMA – Symmetrical dimethyl arginine. L-NMMA – N-monomethyl-L-arginine. CRP – C-reactive protein. NLR – Neutrophil lymphocyte ratio.						

In this analysis, the Nagelkerke R square value was found to be 0.802; thus L-NMMA, CRP and NLR were evaluated as parameters effective in prognosis.

## Discussion

Among routine clinical laboratory parameters, CRP, procalcitonin, IL-6, D-dimer, complete-blood-count parameters (NLR, thrombocyte, haemoglobin), creatinine, liver enzymes (alanine aminotransferase, gamma glutamyltransferase, *etc.*), cardiac troponins are still the most commonly analysed parameters utilized for the prediction of COVID-19 severity (*3*-*9*). In our study, while CRP, D-dimer, fibrinogen, and ferritin were found to be associated with disease severity in the univariate analysis; NLR and CRP were the only routine clinical laboratory parameters determined as independent variates for COVID-19 severity in the multivariate analysis.

The number of studies concerning the involvement of ADMA and associated metabolites in COVID-19 disease is limited in the literature. In their longitudinal study, Hanneman *et al.* showed that ADMA and SDMA could be biomarkers for predicting mortality risk in COVID-19 patients (*19*). In addition to finding a similar correlation for ADMA and SDMA in this study where severe COVID-19 cases demonstrated higher ADMA and SDMA concentrations, we also analysed serum L-NMMA, arginine, and homoarginine concentrations and investigated their correlation with other laboratory and clinical findings and COVID-19 severity. Consequently, we determined that increase in the L-NMMA concentration is an independent factor, and its concentration holds great promise for assessing the severity of the disease.

In literature, the SARS-CoV-2 virus is considered to cause endothelial inflammation and endothelial dysfunction facilitated by NO deficiency. Endothelial damage associated with increases in ADMA, SDMA, and L-NMMA is suggested to play a role in the severity of COVID-19 with a high risk of thrombosis and increased mortality (*24*, *25*). The ADMA, L-NMMA, and SDMA had previously been documented to induce vascular insufficiency and immune response through impairing NO production. The relation of these three biomarkers with COVID-19 severity that is unveiled in this study suggests that ADMA, L-NMMA, and SDMA may participate in COVID-19 physiopathology by triggering vascular insufficiency and immune response. Although studies on the relation of ADMA and SDMA with COVID-19 were previously published, this is the first publication indicating a correlation between L-NMMA concentrations and COVID-19 severity (*19*). Even though L-NMMA concentration is generally 10-fold lower than ADMA, the fact that L-NMMA concentration has a more potent effect on COVID-19 severity may suggest that L-NMMA may contribute to the reduction of NOS enzyme activity at least as potently as ADMA. This is the first study to indicate a potential role for L-NMMA analysis in determining the severity of COVID-19 disease.

In addition to other mechanisms, ADMA, SDMA, and L-NMMA may be associated with various organ dysfunctions and aberrations, such as oxidative stress, cardiovascular thromboembolic events, neurological damage, acute kidney injury, and multiple organ failure, which are relatively common in COVID-19 patients (*26*, *27*).

There are important limitations in the current study, one of which is the limited number of COVID-19 patients accessed during the study period, complicating the evaluation of a complex disease such as COVID-19. The absence of asymptomatic patients and non-hospitalized patients is another limitation of the current study. Since only 2 of the severe cases died in our study group, no specific evaluation was made about them. In addition, NO concentrations of the patients and controls are not known. Along with these, as one of the most important problems of most common COVID-19 studies, asymptomatic cases could not have been included in the study group. Moreover, it should be noted that the drugs (steroid, anti-inflammatory, *etc.*) used by these patients may have significant effects on metabolic pathways, including the arginine/L-NMMA/ADMA/SDMA interactions. It is worth noting that, due to the laborious and time-consuming nature of methylated arginine product quantifications, and the requirement of sophisticated technical infrastructure, their analyses are not routinely performed at every clinical biochemistry laboratory, and measurement of these molecules especially at emergency services are not possible yet. However, it is predicted that widespread use of mass detectors in emergency services or development of immunoassays will allow additional parameters to be routinely serviced soon.

In conclusion, while age, gender, ADMA, SDMA, L-NMMA, NLR, CRP, ferritin, D-dimer, and fibrinogen stood out as risk factors for COVID-19 severity in univariate analysis; only CRP, NLR, and L-NMMA were identified as independent risk factors through multivariate analysis. In light of the results of this study, we suggest that arginine metabolites, especially L-NMMA may have significant roles in the physiopathology of COVID-19 severity.
